# Decreases in COVID-19 Cases, Emergency Department Visits, Hospital Admissions, and Deaths Among Older Adults Following the Introduction of COVID-19 Vaccine — United States, September 6, 2020–May 1, 2021

**DOI:** 10.15585/mmwr.mm7023e2

**Published:** 2021-06-11

**Authors:** Athalia Christie, S. Jane Henley, Linda Mattocks, Robyn Fernando, Amy Lansky, Farida B. Ahmad, Jennifer Adjemian, Robert N. Anderson, Alison M. Binder, Kelly Carey, Deborah L. Dee, Taylor Dias, William M. Duck, Denise M. Gaughan, Brianna Casey Lyons, A.D. McNaghten, Meeyoung M. Park, Hannah Reses, Loren Rodgers, Katharina Van Santen, David Walker, Michael J. Beach

**Affiliations:** 1 CDC COVID-19 Response Team.

Throughout the COVID-19 pandemic, older U.S. adults have been at increased risk for severe COVID-19–associated illness and death (*1*). On December 14, 2020, the United States began a nationwide vaccination campaign after the Food and Drug Administration’s Emergency Use Authorization of Pfizer-BioNTech COVID-19 vaccine. The Advisory Committee on Immunization Practices (ACIP) recommended prioritizing health care personnel and residents of long-term care facilities, followed by essential workers and persons at risk for severe illness, including adults aged ≥65 years, in the early phases of the vaccination program (*2*). By May 1, 2021, 82%, 63%, and 42% of persons aged ≥65, 50–64, and 18–49 years, respectively, had received ≥1 COVID-19 vaccine dose. CDC calculated the rates of COVID-19 cases, emergency department (ED) visits, hospital admissions, and deaths by age group during November 29–December 12, 2020 (prevaccine) and April 18–May 1, 2021. The rate ratios comparing the oldest age groups (≥70 years for hospital admissions; ≥65 years for other measures) with adults aged 18–49 years were 40%, 59%, 65%, and 66% lower, respectively, in the latter period. These differential declines are likely due, in part, to higher COVID-19 vaccination coverage among older adults, highlighting the potential benefits of rapidly increasing vaccination coverage.

CDC analyzed the age distribution of COVID-19 vaccination during December 14, 2020–May 1, 2021. To visualize trends before and after vaccine introduction, rates of reported COVID-19 cases, ED visits, hospitalizations, and deaths by age group are presented for September 6, 2020–May 1, 2021. Daily data about COVID-19 vaccine doses administered in the United States, including partial and full vaccination, were collected by vaccination providers and reported to CDC through multiple sources.* Daily COVID-19 case data were obtained from CDC’s case-based surveillance system^†^ as reported by jurisdictional health departments. Daily ED visits for patients with a diagnosis of COVID-19^§^ (COVID-19 ED visit) were obtained from the National Syndromic Surveillance Program. Daily admissions data on persons newly admitted to a hospital with a laboratory-confirmed COVID-19 diagnosis at the time of admission (COVID-19 hospital admission) were obtained from the U.S. Department of Health and Human Services (HHS) Unified Hospital dataset.^¶^ Weekly COVID-19 death data were collected from CDC’s National Vital Statistics System.** U.S. Census Bureau midyear 2019 population estimates (as of July 1, 2020)^††^ were used to calculate vaccination, case, hospital admission, and death rates per 100,000 population. ED visits were shown as visits with a COVID-19 diagnosis per 100,000 ED visits reported.

To assess differences by age, CDC calculated the weekly proportion, rate, and rate ratio by age group for COVID-19 outcomes, including cases, ED visits, hospital admissions, and deaths.^§§^ Trends were examined by plotting weekly rates by age group and rate ratios comparing persons aged ≥65 years (≥70 years for hospital admissions^¶¶^) with those aged 18–49 years during September 6, 2020–May 1, 2021. Differences in age group–specific average weekly proportions, rates, and rate ratios for COVID-19 outcomes were compared during two periods: November 29–December 12, 2020 (prevaccine) and April 18–May 1, 2021 (most recent data available, accounting for reporting lag); 95% confidence intervals (CIs) and p values for these differences and for rate ratios were constructed by applying the parametric bootstrap method to 10,001 replicate pseudosamples (*3*). Analyses were conducted using R software (version 4.0.0; R Foundation). These activities were reviewed by CDC and were conducted consistent with applicable federal law and CDC policy.***

COVID-19 vaccine administration increased from introduction on December 14, 2020, to a peak 7-day moving average of 3.3 million doses per day in mid-April before decreasing to 2.2 million doses per day by May 1, 2021 (Figure 1). Among persons aged ≥65 years, 25% had received ≥1 vaccine dose by February 6, 2021, 50% by March 3, 2021, and 82% by the end of the analysis period, May 1, 2021 (Figure 1). Among persons aged 18–49 years, 7%, 10%, and 42% had received ≥1 vaccine dose by the same dates, respectively. By May 1, 2021, 69% of persons aged ≥65 years and 26% of persons 18–49 years were fully vaccinated. 

**FIGURE 1:**
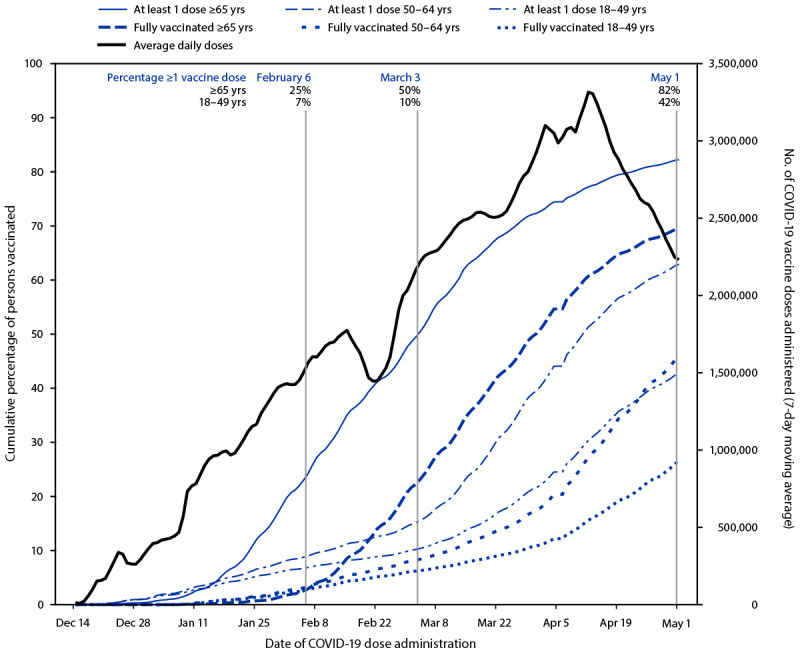
Average daily* number of total COVID-19 vaccine doses administered and cumulative percentage of adults aged ≥18 years who received ≥1 dose and who were fully vaccinated, by age group^†^ — United States,^§^ December 14, 2020–May 1, 2021 * Based on 7-day moving average. ^†^ Age was unknown for 8% of fully vaccinated persons. ^§^ Texas does not report demographic-specific dose number information to CDC, so data for Texas are not represented in cumulative percentage of population vaccinated.

**Sources:** **COVID-19 Vaccination Trends in the United States,** https://data.cdc.gov/Vaccinations/COVID-19-Vaccination-Trends-in-the-United-States-N/rh2h-3yt2 and COVID-19 Vaccination Demographics in the United States, https://data.cdc.gov/Vaccinations/COVID-19- Vaccination-Demographics-in-the-United-St/km4m-vcsb; accessed May 26, 2021.

COVID-19 incidence increased in all age groups during September 6, 2020–January 2, 2021, and then decreased (Figure 2). The weekly rate ratio of COVID-19 incidence among older adults to younger adults was highest in late December and then declined. Compared with the prevaccination period of November 29–December 12, 2020, COVID-19 incidence during April 18–May 1, 2021, was 69% lower among all adults, and 79%, 71%, and 66% lower among persons aged ≥65, 50–64, and 18–49 years respectively (Table). The proportion of COVID-19 cases diagnosed in persons aged ≥65 years decreased from 16.0% to 10.7% (p<0.001). The rate ratio of COVID-19 incidence among persons aged ≥65 years to that among persons aged 18–49 years decreased 40% (p<0.001) from 0.68 (95% CI = 0.67–0.68) to 0.40 (95% CI = 0.40–0.41) (p<0.001).

**FIGURE 2:**
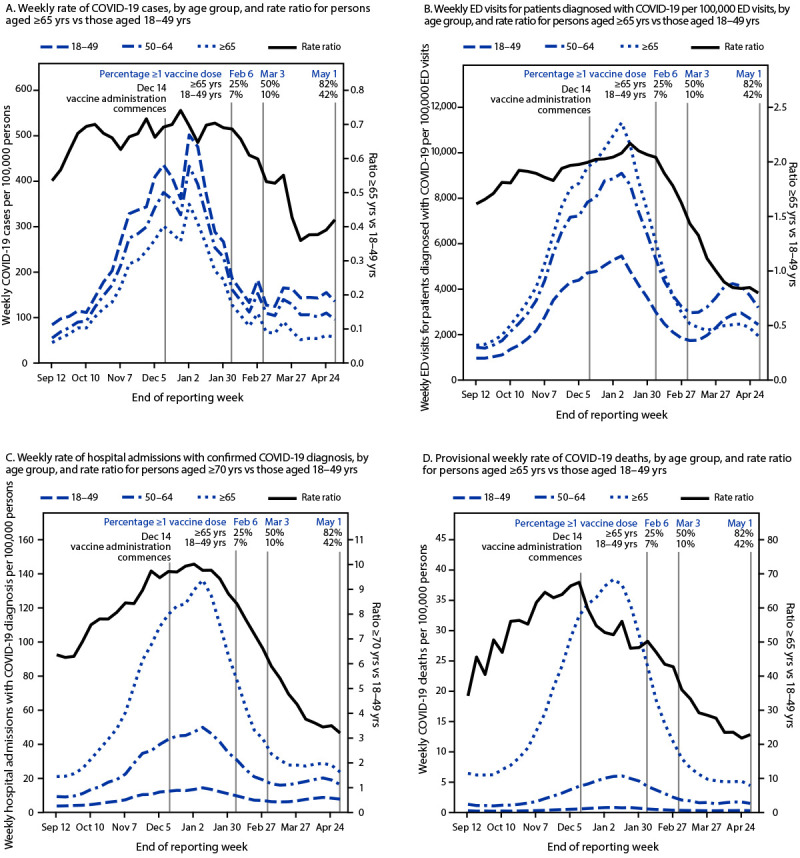
Weekly COVID-19 rates (A),*^,^^†^^,^^§^ emergency department visits for patients with a diagnosis of COVID-19 (B),^¶^ hospital admissions with confirmed COVID-19 diagnosis (C),**^,^^††^ and COVID-19 deaths (D)^§§^^,^^¶¶^ among adults, by age group, and rate ratio for persons aged ≥65 or ≥70 years versus 18–49 years — United States, September 6, 2020–May 1, 2021 **Sources:** CDC’s case-based COVID-19 surveillance system, accessed May 26, 2021 (A); National Syndromic Surveillance Program, accessed May 26, 2021 (B); U.S. Department of Health and Human Services Unified Hospital dataset, accessed May 26, 2021 (C); National Vital Statistics System, accessed May 26, 2021 (D). **Abbreviation:** ED = emergency department. * COVID-19 cases per 100,000 persons. ^†^ Case classifications for COVID-19 are described in https://ndc.services.cdc.gov/case-definitions/coronavirus-disease-2019-2020-08-05 and https://www.cdc.gov/ coronavirus/2019-ncov/covid-data/faq-surveillance.html. ^§^ Demographic data are based on a subset of COVID-19 cases for whom case-level data have been reported by state and territorial jurisdictions, accounting for approximately 80% of all cases reported to CDC. Patient age was unknown for 0.7% of cases. ^¶^ ED visits are shown as visits for patients with a diagnosis of COVID-19 per 100,000 ED visits reported. ED visits for patients with a diagnosis of COVID-19 are defined as ED visits with any of the following: *International Classification of Diseases, Tenth Revision* codes U07.1 or J12.82 or Systematized Nomenclature of Medicine 840539006, 840544004, or 840533007. Patient age was unknown for 0.4% of ED visits. ** Hospital admissions with confirmed COVID-19 diagnosis per 100,000 persons. ^††^ Dataset includes data reported by hospitals registered with the Centers for Medicare & Medicaid Services. Data were reported to the U.S. Department of Health and Human Services directly from facilities or via a state submission; on May 1, 2021, 98.5% of hospitals reported. This analysis includes Veterans Administration, Defense Health Agency, and Indian Health Services hospitals and excludes psychiatric, rehabilitation, and religious nonmedical hospitals. Patient age was unknown for 4% of hospital admissions. ^§§^ COVID-19 deaths per 100,000 persons. ^¶¶^ Deaths with confirmed or presumed COVID-19 as an underlying or contributing cause of death, with *International Classification of Diseases, Tenth Revision* code U07.1. Provisional data are incomplete. Decedent age was unknown for <0.01% of deaths.

**TABLE:** Number, proportion, rate,* and rate ratio of COVID-19 cases,^†^ emergency department visits for patients with a diagnosis of COVID-19,^§^ hospital admissions with a confirmed COVID-19 diagnosis, and COVID-19 deaths^¶^ among adults aged ≥18 years, by age group, for selected 2-week periods — United States, November 29–December 12, 2020, and April 18–May 1, 2021

Period, COVID-19 outcome, and age group (yrs)	Average weekly no. (% by age group)	Average weekly outcome per 100,000	Rate ratio comparing older age groups with age 18–49 yrs (95% CI) **	Change from November 29–December 12, 2020 to April 18–May 1, 2021		
				Absolute change in proportion	Relative change in rate, %	Relative change in rate ratio, %
**November 29–December 12, 2020 (prevaccine administration)**						
**Cases**	**964,697 (100.0)**	**378**	N/A	N/A	N/A	N/A
≥65	154,829 (16.0)	286	0.68 (0.67–0.68)	N/A	N/A	N/A
50–64	225,715 (23.4)	359	0.85 (0.85–0.85)	N/A	N/A	N/A
18–49	584,154 (60.6)	423	1.0 (referent)	N/A	N/A	N/A
**ED visits**	**108,689 (100.0)**	**6,409**	N/A	N/A	N/A	N/A
≥65	41,208 (37.9)	9,008	1.99 (1.96–2.01)	N/A	N/A	N/A
50–64	28,537 (26.3)	7,513	1.66 (1.64–1.68)	N/A	N/A	N/A
18–49	38,945 (35.8)	4,536	1.0 (referent)	N/A	N/A	N/A
**Hospital admissions**	**90,349 (100.0)**	**35**	N/A	N/A	N/A	N/A
≥70	41,178 (45.6)	112	9.60 (9.45–9.76)	N/A	N/A	N/A
50–69	32,976 (36.5)	41	3.50 (3.45–3.56)	N/A	N/A	N/A
18–49	16,195 (17.9)	12	1.0 (referent)	N/A	N/A	N/A
**Deaths**	**19,666 (100.0)**	**7.7**	N/A	N/A	N/A	N/A
≥65	16,557 (84.2)	30.6	66.93 (62.11–72.29)	N/A	N/A	N/A
50–64	2,477 (12.6)	3.9	8.60 (7.92–9.38)	N/A	N/A	N/A
18–49	633 (3.2)	0.5	1.0 (referent)	N/A	N/A	N/A
**April 18–May 1, 2021 (most recent data available at time of report, allowing time for reporting lag)**						
**Cases**	**297,618 (100.0)**	**117**	N/A	N/A	−69^††^	N/A
≥65	31,802 (10.7)	59	0.40 (0.40–0.41)	−5.4^††^	−79^††^	−40^††^
50–64	64,796 (21.8)	103	0.71 (0.70–0.71)	−1.6^††^	−71^††^	−17^††^
18–49	201,021 (67.5)	145	1.0 (referent)	7.0^††^	−66^††^	N/A
**ED visits**	**46,308 (100.0)**	**2,628**	N/A	N/A	−59^††^	N/A
≥65	9,580 (20.7)	2,093	0.82 (0.80–0.84)	−17.2^††^	−77^††^	−59^††^
50–64	13,449 (29.0)	3,437	1.35 (1.33–1.37)	2.8^††^	−54^††^	−19^††^
18–49	23,280 (50.3)	2,550	1.0 (referent)	14.4^††^	−44^††^	N/A
**Hospital admissions**	**33,600 (100.0)**	**13**	N/A	N/A	−63^††^	N/A
≥70	9,260 (27.6)	25	3.33 (3.26–3.41)	−18.0^††^	−78^††^	−65^††^
50–69	13,850 (41.2)	17	2.27 (2.22–2.32)	4.7^††^	−58^††^	−35^††^
18–49	10,490 (31.2)	8	1.0 (referent)	13.3^††^	−35^††^	N/A
**Deaths**	**3,918 (100.0)**	**1.5**	N/A	N/A	−80^††^	N/A
≥65	2,663 (68.0)	4.9	22.43 (20.17–25.18)	−16.2^††^	−84^††^	−66^††^
50–64	952 (24.3)	1.5	6.89 (6.12–7.82)	11.7^††^	−62^††^	−20
18–49	304 (7.7)	0.2	1.0 (referent)	4.5^††^	−52^††^	N/A

**Sources**: CDC’s case-based COVID-19 surveillance system, National Syndromic Surveillance Program, U.S. Department of Health and Human Services Unified Hospital dataset, National Vital Statistics System; accessed May 26, 2021.

**Abbreviations:** CI = confidence interval; ED = emergency department; ICD-10 = *International Classification of Diseases, Tenth Revision*; N/A = not applicable.  * COVID-19 cases, hospital admissions with confirmed COVID-19 diagnosis, and COVID-19 deaths per 100,000 persons and ED visits for patients with a diagnosis of COVID-19 per 100,000 ED visits.

^†^ The case classifications for COVID-19 are described in an updated interim COVID-19 position statement and case definition issued by the Council of State and Territorial Epidemiologists on August 5, 2020 (https://ndc.services.cdc.gov/case-definitions/coronavirus-disease-2019-2020-08-05). However, some variation in how jurisdictions implement these case classifications was observed. More information on how CDC collects COVID-19 case surveillance data can be found at https://www.cdc.gov/coronavirus/2019-ncov/covid-data/faq-surveillance.html.

^§^ ED visits for COVID-19 are defined as ED visits with any of the following: ICD-10 codes U07.1 or J12.82 or Systematized Nomenclature of Medicine codes 840539006, 840544004, or 840533007.

^¶^ Deaths with confirmed or presumed COVID-19 as an underlying or contributing cause of death with ICD-10 code U07.1. Provisional data are incomplete. Data from May 2021 are less complete because of reporting lags.

** CIs and p values were constructed using the parametric bootstrap method using 10,001 replicate pseudosamples. CIs were formed using the quantiles of the bootstrap distributions, and p values were based on the proportion of pseudosample values below the 0.025 or above the 0.975 quantile.

^††^ The change in measure from November 29–December 12, 2020, to April 18–May 1, 2021, was statistically significantly different (p<0.001).

During September 6, 2020–May 1, 2021, COVID-19 ED visits per 100,000 ED visits peaked among all age groups during the week of January 3–January 9, 2021, approximately 1 week after the peak in incidence (Figure 2). The weekly rate ratio of COVID-19 ED visits among older adults to younger adults was highest in mid-January and then declined. Compared with the prevaccination period of November 29–December 12, 2020, COVID-19 ED visits per 100,000 ED visits during April 18–May 1, 2021, were 59% lower among all adults, with a larger change for persons aged ≥65 years (77%) than for other age groups (Table). During November 29–December 12, 2020, and April 18–May 1, 2021, persons aged ≥65 years accounted for 37.9% and 20.7%, respectively, of adult COVID-19 ED visits. The rate ratio of COVID-19 ED visits per 100,000 ED visits among persons aged ≥65 years to those among persons aged 18–49 years decreased 59% (p<0.001) from 1.99 (95% CI = 1.96–2.01) to 0.82 (95% CI = 0.80–0.84).

Rates of COVID-19 hospital admissions peaked during the week of January 3–January 9, 2021, approximately 1 week after case incidence peaked (Figure 2). The trend in the weekly rate ratio of COVID-19 hospital admissions among older adults to younger adults followed a similar pattern as ED visits. Compared with hospital admissions during the prevaccination period of November 29–December 12, 2020, adult COVID-19 hospital admissions rates were 63% lower among all adults, with the largest change (78%) among adults aged ≥65 years, during April 18–May 1, 2021. Although COVID-19 admissions remained highest among persons aged ≥70 years, the proportion of adult COVID-19 hospital admissions among this age group decreased from 45.6% during November 29–December 12, 2020, to 27.6% during April 18–May 1, 2021 (p<0.001) (Table). The rate ratio of COVID-19 hospital admission rates among persons aged ≥70 years to those among persons aged 18–49 years decreased 65% (p<0.001) from 9.60 (95% CI = 9.45–9.76) to 3.33 (95% CI = 3.26–3.41) (p<0.001).

During September 6, 2020–May 1, 2021, weekly COVID-19 death rates peaked between January 3–January 16, 2021, among all age groups and then decreased through May 1, 2021 (Figure 2). The weekly rate ratio of COVID-19 deaths among older adults to those among younger adults was highest in mid-December and then declined. Mortality remained highest for persons aged ≥65 years; however, the proportion of COVID-19 deaths that occurred among this age group decreased from 84.2% during the prevaccination period of November 29–December 12, 2020, to 68.0% during April 18–May 1, 2021 (p<0.001) (Table). The rate ratio of COVID-19 death rates among persons aged ≥65 years to those among persons aged 18–49 years decreased 66% (p<0.001) from 66.93 (95% CI = 62.11–72.29) to 22.43 (95% CI = 20.17–25.18).

## Discussion

Weekly COVID-19 incidence among adults increased during September 6, 2020–January 2, 2021. After this peak, incidence, followed by rates of ED visits, hospital admissions, and deaths declined among all adult age groups. During September 6–December 14, 2020, before the commencement of vaccine administration, the rate ratios of COVID-19 outcomes among older adults to younger adults were either stable or increasing. The ratio for COVID-19 deaths began to decline in mid-December while rate ratios for COVID-19 incidence, ED visits, and hospital admissions began to decline in late December to mid-January. Comparing the 2-week prevaccination period with 2 weeks in late April, declines were significantly greater among older adults, who had higher vaccination coverage, than among younger adults, who had lower coverage. These age-stratified results provide ecologic evidence of the likely contribution of vaccination coverage to reducing COVID-19 outcomes.

These data are consistent with other preliminary reports showing a reduction in COVID-19 cases and severe illness in populations with high vaccination coverage. An ecologic study from Israel found the ratio of COVID-19 patients aged ≥70 years requiring mechanical ventilation to those aged <50 years declined 67% within 3 months of a nationwide vaccination campaign prioritizing persons aged >60 years (*4*). In separate studies analyzing Israeli surveillance data, COVID-19 incidence, hospitalizations, and deaths markedly declined across all age groups as cumulative vaccination coverage increased (*5*), and vaccine effectiveness of 46% for COVID-19 infection, 74% for hospitalization, and 72% for death, was observed during 14–20 days after the first dose (*6*). A CDC evaluation at 24 hospitals found that receipt of COVID-19 vaccine was 64% effective against COVID-19 hospitalization among partially vaccinated adults aged ≥65 years and 94% effective among fully vaccinated adults aged ≥65 years (*7*).

The findings in this report are subject to at least five limitations. First, this was an ecologic analysis based on aggregated data that does not account for variability in reporting or vaccination coverage among jurisdictions, between rural and urban areas, or by race and ethnicity. Second, states and territories adapted ACIP recommendations (*8*); therefore, the populations eligible and timing of each vaccination phase varied across jurisdictions. Third, the case, ED, and hospital data are subsets of total outcomes, and all data are subject to reporting inconsistencies and delays. Fourth, the analysis does not account for concomitant effects, including the spread of more transmissible SARS-CoV-2 variants, the general surge and subsequent decline in COVID-19 cases, the use of recommended therapeutics (*9*), and the implementation and relaxation of community-level prevention policies in individual jurisdictions. However, by analyzing the relative changes in ratios comparing rates between older and younger age groups, these results were less likely to be influenced by population effects that might have affected all age groups similarly. Finally, no attempt was made to quantify the percentage of these differential rate ratio changes that were potentially attributable to vaccination. The decline in the rate ratio for deaths between older and younger adults, for example, began just after vaccine introduction; therefore, vaccine coverage can account for only part of the decline. Time trend analyses, and other analytic approaches, might enhance understanding of the impact of vaccination on population-level dynamics.

From November 29, 2020, to May 1, 2021, COVID-19 incidence, ED visits, hospital admissions, and deaths declined more in older adults, who had higher vaccination coverage, than in younger adults, who had lower coverage. Despite sufficient vaccine supply and expanding eligibility, administration of COVID-19 vaccines has steadily declined in adults since mid-April 2021. These results suggest that tailored efforts by state and local jurisdictions to rapidly increase vaccine coverage among all eligible age groups could contribute to further reductions in COVID-19 cases and severe outcomes. Such efforts include effectively communicating the benefits of vaccination, ensuring equitable access and convenience, empowering trusted messengers, including primary health care providers, and engaging communities.

**Summary**

**What is already known about this topic?**

COVID-19 vaccination began in the United States in December 2020, and adults aged ≥65 years were prioritized in early phases.

**What is added by this report?**

By May 1, 2021, 82%, 63%, and 42% of adults aged ≥65, 50–64, and 18–49 years, respectively, had received ≥1 vaccine dose. From November 29–December 12, 2020 to April 18–May 1, 2021, the rate ratios of COVID-19 incidence, emergency department visits, hospital admissions, and deaths among adults aged ≥65 years (≥70 years for hospitalizations) to adults aged 18–49 years declined 40%, 59%, 65%, and 66%, respectively.

**What are the implications for public health practice?**

The greater decline in COVID-19 morbidity and mortality in older adults, the age group with the highest vaccination rates, demonstrates the potential impact of increasing population-level vaccination coverage.

## Footnotes

* COVID-19 vaccine administration data are reported to CDC by multiple entities using immunization information systems, the Vaccine Administration Management System, pharmacy systems, or direct submission of electronic health records. (https://www.cdc.gov/coronavirus/2019-ncov/vaccines/distributing/about-vaccine-data.html). Persons were considered fully vaccinated if they received the second dose in a 2-dose COVID-19 vaccine series (Pfizer-BioNTech or Moderna) or 1 dose of the single-dose Janssen (Johnson and Johnson) COVID-19 vaccine.

† CDC official counts of COVID-19 cases and deaths, released daily at https://covid.cdc.gov/covid-data-tracker, are aggregate counts from reporting jurisdictions. Some jurisdictions electronically submit standardized information for individual cases of COVID-19 to CDC via a case report form developed for the CDC COVID-19 response (https://www.cdc.gov/coronavirus/2019-ncov/php/reporting-pui.html) or via the CDC National Notifiable Diseases Surveillance System (https://www.cdc.gov/nndss/action/covid-19-response.html). Individual-level case report data were available for approximately 80% of the aggregate number of confirmed cases.

§ The National Syndromic Surveillance Program collects electronic health data, including ED visits with confirmed COVID-19 diagnoses, from a subset of hospitals in 49 states (all but Hawaii) and the District of Columbia (71% of nonfederal EDs in the United States). ED visits for COVID-19 are defined as ED visits with any of the following: *International Classification of Diseases, Tenth Revision* codes U07.1 or J12.82 or Systematized Nomenclature of Medicine codes 840539006, 840544004, or 840533007. https://www.cdc.gov/nssp/overview.html

¶ The HHS Unified Hospital dataset includes data reported by hospitals registered with the Centers for Medicare & Medicaid Services. Data, including counts of new hospital admissions of patients with confirmed COVID-19 by age group, are reported to HHS either directly from facilities or via a state submission; on May 1, 2021, 98.5% of hospitals reported data. This analysis includes Veterans Administration, Defense Health Agency, and Indian Health Services hospitals and excludes psychiatric, rehabilitation, and religious nonmedical hospitals. https://www.hhs.gov/sites/default/files/covid-19-faqs-hospitals-hospital-laboratory-acute-care-facility-data-reporting.pdf

** COVID-19 deaths include deaths for which COVID-19 was listed on the death certificate as a confirmed or presumed underlying cause of death or contributing cause of death (ICD-10 code U07.1). https://www.cdc.gov/nchs/nvss/vsrr/covid19/tech_notes.htm

††
https://www.census.gov/data/tables/time-series/demo/popest/2010s-national-detail.html

§§ Patient age was unknown for 8% of vaccinated persons, 0.7% of cases, 0.4% of ED visits, 4% of hospital admissions, and <0.01% of deaths.

¶¶ Hospital admissions were submitted by predefined age group (<18 years, 18–19 years, 10-year age groups from 20–79 years, and ≥80 years) and could not be aggregated from single year of age as was done for cases, ED visits, and deaths.

*** 45 C.F.R. part 46, 21 C.F.R. part 56; 42 U.S.C. Sect. 241(d); 5 U.S.C. Sect. 552a; 44 U.S.C. Sect. 3501 et seq.

## References

[1] Bialek S, Boundy E, Bowen V, ; CDC COVID-19 Response Team. Severe outcomes among patients with coronavirus disease 2019 (COVID-19)—United States, February 12–March 16, 2020. MMWR Morb Mortal Wkly Rep 2020;69:343–6. 10.15585/mmwr.mm6912e2 32214079 PMC7725513

[2] Dooling K, Marin M, Wallace M, The Advisory Committee on Immunization Practices’ updated interim recommendation for allocation of COVID-19 vaccine—United States, December 2020. MMWR Morb Mortal Wkly Rep 2021;69:1657–60. 10.15585/mmwr.mm695152e2 33382671 PMC9191902

[3] Efron B, Tibshirani R. Bootstrap methods for standard errors, confidence intervals, and other measures of statistical accuracy. Stat Sci 1986;1:54–75. 10.1214/ss/1177013815

[4] Rinott E, Youngster I, Lewis YE. Reduction in COVID-19 patients requiring mechanical ventilation following implementation of a national COVID-19 vaccination program—Israel, December 2020–February 2021. MMWR Morb Mortal Wkly Rep 2021;70:326–8. 10.15585/mmwr.mm7009e3 33661863 PMC7948930

[5] Haas EJ, Angulo FJ, McLaughlin JM, Impact and effectiveness of mRNA BNT162b2 vaccine against SARS-CoV-2 infections and COVID-19 cases, hospitalisations, and deaths following a nationwide vaccination campaign in Israel: an observational study using national surveillance data. Lancet 2021;397:1819–29. 10.1016/S0140-6736(21)00947-8 33964222 PMC8099315

[6] Dagan N, Barda N, Kepten E, BNT162b2 mRNA Covid-19 vaccine in a nationwide mass vaccination setting. N Engl J Med 2021;384:1412–23. 10.1056/NEJMoa2101765 33626250 PMC7944975

[7] Tenforde MW, Olson SM, Self WH, ; IVY Network; HAIVEN Investigators. Effectiveness of Pfizer-BioNTech and Moderna vaccines against COVID-19 among hospitalized adults aged ≥65 Years—United States, January–March 2021. MMWR Morb Mortal Wkly Rep 2021;70:674–9. 10.15585/mmwr.mm7018e1 33956782 PMC9368749

[8] Raifman J, Nocka K, Jones D, COVID-19 US state policy database. Boston, MA: Boston University School of Public Health; 2020. http://www.tinyurl.com/statepolicies

[9] COVID-19 Treatment Guidelines Panel. Coronavirus disease 2019 (COVID-19) treatment guidelines. Bethesda, MD: National Institutes of Health; 2021. https://www.covid19treatmentguidelines.nih.gov/34003615

